# Online Activity Recognition Combining Dynamic Segmentation and Emergent Modeling

**DOI:** 10.3390/s22062250

**Published:** 2022-03-14

**Authors:** Zimin Xu, Guoli Wang, Xuemei Guo

**Affiliations:** 1 School of Computer Science and Engineering, Sun Yat-sen University, Guangzhou 510006, China; xuzm23@mail2.sysu.edu.cn (Z.X.); guoxuem@mail.sysu.edu.cn (X.G.); 2 Key Laboratory of Machine Intelligence and Advanced Computing, Ministry of Education, Guangzhou 510006, China

**Keywords:** online activity recognition, dynamic segmentation, emergent modeling, directed weighted network

## Abstract

Activity recognition is fundamental to many applications envisaged in pervasive computing, especially in smart environments where the resident’s data collected from sensors will be mapped to human activities. Previous research usually focuses on scripted or pre-segmented sequences related to activities, whereas many real-world deployments require information about the ongoing activities in real time. In this paper, we propose an online activity recognition model on streaming sensor data that incorporates the spatio-temporal correlation-based dynamic segmentation method and the stigmergy-based emergent modeling method to recognize activities when new sensor events are recorded. The dynamic segmentation approach integrating sensor correlation and time correlation judges whether two consecutive sensor events belong to the same window or not, avoiding events from very different functional areas or with a long time interval in the same window, thus obtaining the segmented window for every single event. Then, the emergent paradigm with marker-based stigmergy is adopted to build activity features that are explicitly represented as a directed weighted network to define the context for the last sensor event in this window, which does not need sophisticated domain knowledge. We validate the proposed method utilizing the real-world dataset Aruba from the CASAS project and the results show the effectiveness.

## 1. Introduction

The great progress of ubiquitous computing has contributed to the rapid development of various sensors that are usually used to collect information of interest. When combined with efficient machine learning or deep learning techniques, the collected information is very important for the development of a wide range of applications. One of the application areas is the smart home environment, in which human and environmental information is adopted to track the functional condition of interested objects. The aging population [1], the healthcare costs [2] and the desire for aging in place [3] highlight the necessity of developing these technologies. In order to live a functionally independent life, residents must have the ability to complete activities of daily living (ADLs), such as eating, bathing, etc. Therefore, it is crucial to automatically recognize and track the ADLs of the interested objects for monitoring their functional status.

A range of activity recognition (AR) technologies are very effective in scripted or pre-segmented sequences of activity. However, the actual deployment of some real-world scenarios requires continuous AR on streaming sensor data. In the context of providing timely and proactive assistance (such as prompting systems [4,5]), AR on streaming/online sensor data is required to know which tasks the resident is currently performing and estimate whether the individual is competent for the task. Only in this way can suitable intervention be implemented and proper help be provided. The task of online AR is non-trivial, because it is usually unable to obtain the data fully describing the activity in this case, and the algorithm must determine the activity being executed according to the partially observed data and other context information [6]. To provide an accurate online AR, data segmentation and feature extraction are two crucial steps, which are important factors able to decrease or increase the performance of an AR model.

The online and continuous methods classify every single sensor event on the basis of the context information encoded by the preceding sensor events in the sliding window, where the window size has to be chosen adequately. There are two strategies for streaming data segmentation: fixed and dynamic window sizes. The former divides the entire sensor stream into a series of sliding windows that have the same time interval or the same number of sensor events. Such a method is convenient to operate, but the shortcomings are that it cannot intuitively reflect the actual occurrence of the activities and the inappropriate choice of the window size (too short or too long) will result in poor performance. For instance, two or more activities may be covered in a sliding window, or the sensor events corresponding to one activity are divided into several windows. The latter dynamic method determines the segmentation points in a more flexible way, and it has been proven to achieve better results than the static method [7]. Therefore, we propose a dynamic streaming sensor data segmentation approach incorporating sensor correlation and time correlation, which can avoid placing sensor events with weak spatio-temporal correlation in the same sliding window.

After segmentation, extracting advisable features served as the basis of classifier from the segmented window is of great significance for the task of recognizing activities. The traditional activity modeling methods are often based on the statistical characteristics [8] and frequency-domain features [9,10]. These features have been demonstrated to be valuable for AR; however, a lot of hidden activity information is ignored and sophisticated domain knowledge is needed. The question of how to fully extract features composed of information related to behavioral semantics and spatio-temporal characteristics, and establish an effective representation to characterize the activity information, is a difficulty for AR. Furthermore, the different ways of completing a type of activity and the overlapping functional areas between different activities will lead to the misclassification of activities, which requires solving the confusion between activities. We propose a stigmergy-based emergent modeling method [11,12], and the directed-weighted network (DWN) is used as the explicit representation of the extracted features.

In this work, we propose an online AR framework on streaming discrete binary sensor data, which integrates a dynamic streaming sensor data segmentation method, emergent modeling method and deep learning technology. Specifically, the dynamic segmentation approach derives the appropriate window size for each individual sensor event when it is recorded, and ensures that the temporal and spatial correlation between the preceding sensor data and the last event of interest in a sliding window is above the corresponding threshold. The emergent computing paradigm with marker-based stigmergy and DWN are employed to extract activity features and explicitly represent features, respectively. Finally, the combination of a convolutional neural network and long short-term memory network (CNN-LSTM) realizes the task of identifying ongoing activities at a fine-grained level. We employ the fully annotated dataset Aruba collected by the Center for Advanced Studies in Adaptive Systems (CASAS) project [13] to evaluate our framework. The main contributions are as follows:

- The dynamic streaming sensor data segmentation approach incorporating sensor correlation and time correlation can reduce the probability of sensor events with large time intervals or from very different functional areas in the same sliding window, so as to weaken their influence on the context information defining the last sensor event.
- By explicitly representing the activity features extracted based on the emergent computing paradigm in the form of the directed-weighted network, the spatio-temporal characteristics can be embodied without the need of sophisticated domain knowledge, the context information defining the last event in the window can be reflected, and the ambiguity between ADLs can be relieved.

The remainder of this paper is organized as follows: The related works are summarized in Section 2, and the proposed online AR framework that integrates the dynamic segmentation method and the emergent modeling method is elaborated in Section 3. Section 4 presents the experimental results. Finally, the conclusions are presented in Section 5.

## 2. Related Works

Activity recognition plays an important role in people’s real lives, because it can learn in-depth knowledge of human activity from raw data collected from a variety of sensors. There exist a number of methods for AR, which vary according to the underlying sensing technologies responsible for collecting the activity data, and various algorithms that are employed to model and classify activities.

The progress of ubiquitous computing has witnessed the development of a variety of sensors that can be utilized to gather information about human activities. There exist two kinds of monitoring systems: vision-based monitoring and sensor-based monitoring. The vision-based monitoring systems employ visual sensing facilities to detect the behaviors of interested objects and changes in environment [14]. It is difficult to deploy them in the context of smart homes to monitor residents’ ADLs for a long time considering the problem of privacy invasion. The sensor-based AR adopts the sensor network techniques to achieve the purpose of activity monitoring, and it mainly focuses on two approaches: wearable [15,16] and dense sensor-based [17,18] monitoring. Wearable sensors such as accelerometers are generally adopted to identify simple activities defined by ambulatory movements, such as walking, running and sitting. This monitoring method suffers from the issues of the willingness to wear, viability and ability to use, battery life and ease of use. Meanwhile, the dense sensing-based AR embeds sensors (such as passive infrared sensors (PIRs)) within environments to gather information about a more common series of ADLs, such as cooking and sleeping, and it is more suitable for real-life long-term monitoring. Dense sensors can monitor the resident’s motions and environmental parameters so that assistive personnel can deduce the ongoing ADL according to sensor observations, so as to provide timely context-aware assistance. Some ADLs occur in specific functional areas and lead to unique interactions with objects, and researchers have explored the usage of PIR for reflecting the interaction between residents and the environment, thus achieving the goal of AR. For example, Machot et al. propose a windowing algorithm and several statistical spatio-temporal features to identify complex ADLs for multi-user testbeds employing the CASAS dataset and HBMS dataset [19]. Tan et al. elaborate a method that concatenates external features and extracted features and uses a bi-directional LSTM to recognize ADLs using the CASAS dataset [20]. The dataset for the experiments in our work employs binary discrete PIR sensors that are convenient for deployment in a smart home environment and can protect privacy.

There are already plenty of machine learning algorithms applied to AR, which elicit activity models from pre-existing datasets. Different techniques and tools are investigated, such as hidden Markov models (HMM) [21,22,23], dynamic Bayes nets [21], naive Bayes [24], nearest neighbor [25], support vector machines (SVM) [26], conditional random fields (CRF) [27,28] and multiple eigenspaces [29]. These classical pattern recognition methods have made great progress in AR; however, they still have some shortcomings. Feature extraction is usually carried out in a heuristic or hand-crafted manner, which is highly dependent on human experience or domain knowledge, and can only capture shallow features based on human expertise.

With the rapid development of deep learning technology in recent years, the above limitations have been overcome to a certain extent. The deep neural networks commonly used for AR are CNN, deep belief network (DBN) and recurrent neural network (RNN), including its variations, such as LSTM and gated recurrent unit (GRU). To establish an excellent AR system that can provide better classification and prediction performance, Abdellaoui and Douik propose a method in a two-phase recognition system paradigm, which introduces DBN [30]. Tan et al. employ location-based stigmergy for the emergent representation of ADLs, and then integrate it with CNN to complete the task of recognition. Its advantage is that there is no need for any complex domain model when studying and understanding ADLs [17]. Mohmed et al. present an enhanced fuzzy finite state machine (FFSM) model via fusing the traditional FFSM with LSTM and CNN, respectively, to model and recognize ADLs, and evaluate it on a real dataset that they collected and the Aruba dataset [31]. Mutegeki and Han propose a spatially and temporally deep architecture CNN-LSTM that not only improves the prediction performance but also decreases the complexity of the model [32]. With the deep learning models, the feature extraction and model construction are often carried out at the same time. Moreover, the features can be learned automatically and high-level representation can be extracted in the deep layer. In our work, we employ an holistic deep learning-based structure CNN-LSTM to achieve the goal of online AR.

Though well researched, most learning models only use pre-segmented datasets. However, human activities should be monitored in real time in a lot of real-world scenes. This requires the AR algorithm to keep away from pre-segmented sensor data and concentrate on streaming data. Research works in this area are relatively fewer. Krishnan and Cook present several sliding window-based approaches processing streaming data and propose five kinds of fixed-size windowing methods with different weighting factors [33]. In addition, they introduce a dynamic windowing approach, which employs a probabilistic method to dynamically determine the window size. Chen et al. describe a knowledge-driven method that adopts domain knowledge and ontologies extensively to solve the problem of real-time AR [34]. Okeyo et al. present a dynamic sensor data segmentation approach based on the sliding window techniques [35]. Their study explores two types of scenes, overlapping and non-overlapping time windows. Sfar and Bouzeghoub propose another dynamic streaming sensor data segmentation method, which integrates statistical learning and semantic analysis to study the input event sequence and select the more appropriate time-window size, so as to achieve the purpose of dynamic adaptation [36]. The work presented in this paper proposes a dynamic sensor data segmentation method integrating sensor correlation and time correlation and performs online AR on streaming data.

## 3. Online Activity Recognition Framework

**Figure 1:**
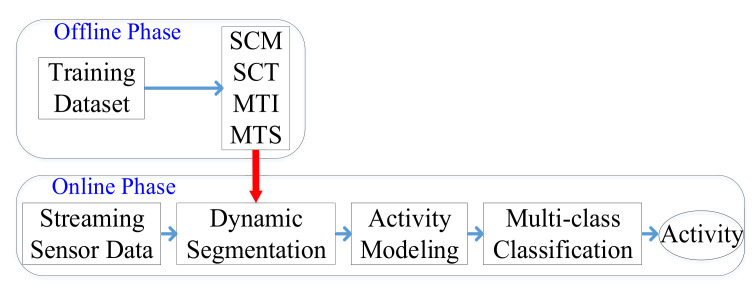
The flow diagram of the online AR framework.

This section discusses the online activity recognition framework integrating the spatio-temporal correlation-based dynamic segmentation method and the stigmergy-based emergent modeling method that provides the basis for the fine-grained recognition algorithm CNN-LSTM. The goal of the proposed method is to classify each individual sensor event with a corresponding activity label as best as possible. The whole process comprises two phases. The first is the offline phase, in which the labeled training data are used to calculate the sensor correlation matrix (SCM), sensor correlation threshold (SCT), maximum time interval (MTI) and maximum time span (MTS). The second is the online phase, the goal of which is to establish the corresponding segmented windows when new sensor events occur based on sensor correlation and time correlation. After completing the above steps in the online phase, the activity modeling step is performed on the segmented windows, followed by the fine-grained multi-class recognition model, which obtains the category of the activity being executed. Figure 1 shows the overall structure of the proposed online AR framework.

### 3.1. Dynamic Streaming Sensor Data Segmentation

Many existing studies use wearable and/or smartphone sensors to implement online activity recognition. Accelerometer-based recognition is relatively easy because such sensors continuously produce data at a fixed frequency, making it possible to segment the entire sensor sequence on the grounds of the time interval or the number of sensor events. The activities identified in these works are often low-level and simple, such as walking, standing and sitting. In comparison, in the context of the smart home, embedded sensors with different sampling rates depending on human activity usually generate data in a discrete way, so there are still problems in dynamic segmentation. In addition, the recognized activities composed of many sub-activities are usually complex, in which it is difficult to obtain the exact boundary and duration of segmentation.

**Figure 2:**
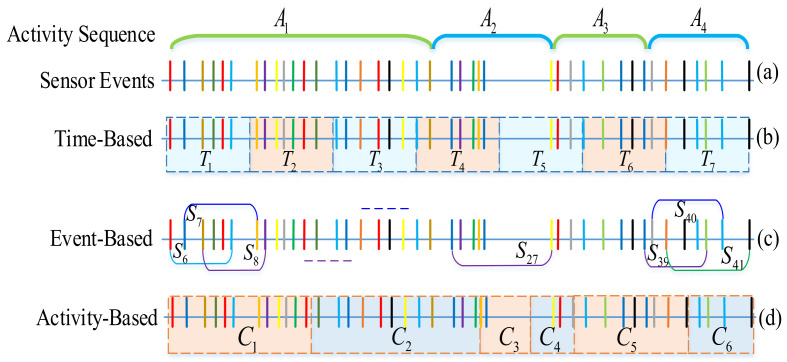
Different segmentation methods on streaming data. (**a**) Ground truth of a sequence of activities. (**b**) Time-based windowing. (**c**) Sensor event-based windowing. (**d**) Activity-based windowing.

Sliding window technology is still the main means for streaming sensor data segmentation, and has been widely used in a lot of applications. Specifically, some common approaches for processing streaming sensor data are presented in Figure 2. Figure 2a shows the ground truth of a series of activities denoted as $A_{1},\;A_{2},\;A_{3},\;A_{4}$, and the relevant sensor events are displayed in chronological order, in which the time interval between each pair of sensor events can be different.

For time-based windowing as displayed in Figure 2b, if a smaller duration is selected, the window may contain insufficient information to make appropriate decisions (or build the model correctly in the training phase). Conversely, if the duration is too long, it is possible to embed information of multiple activities in one window. Therefore, compared with other activities, the activities dominating the window will be more representative, which will seriously affect decision-making. Furthermore, in the case that sensors have an inconstant sampling rate that depends on human motion, there may be no sensor data in some windows.

In terms of sensor event-based windowing, as shown in Figure 2c, the displayed sensor event windows are obtained by taking the sliding window technique with a length of 6 events and a sliding step of one event, whose window duration varies obviously. During the performance of the activity, multiple sensors can be activated, whereas during the silent period, the number of sensor triggers will be reduced. The context of the last sensor event in the segmented window is defined by the events preceding the last one. This method may lead to the relevance between the sensor data in the window and the last sensor event being weak, such as large time intervals or very different functional areas.

Another method is to divide the sensor events into fragments that coincide with the occurrence of each activity, as shown in Figure 2d. Such a method can accurately determine the boundary of segmentation. Nevertheless, one of the implicit but very critical drawbacks is that it must wait for future data before making decisions on past data, i.e., it takes longer to receive enough information to define a segment. In addition, the difficulty lies in how to decide whether two consecutive sensor events belong to the same activity or not. Therefore, we employ a dynamic segmentation method when new sensor events occur in the ambient assisted living environment, which incorporates sensor correlation and time correlation.

#### 3.1.1. Sensor Correlation

Smart environments are usually embedded with a lot of sensors that generate sensor events along the timeline. Generally, the sensor event sequence can be expressed as $\left\{E_{1},\;E_{2},\ldots ,E_{N}\right\}$, where $E_{i}$ represents the *i*th sensor event encoded with template {date,time,sensorID,sensorValue}. Therefore, one of the difficulties is how to determine the preceding sensor data that describe the context for the latest sensor event whenever it is recorded. On the one hand, the dynamic sensor event segmentation method is achieved by calculating the sensor correlation measured by the mutual information between sensors in this work. Mutual information is usually defined by the interdependence of two random variables. In the current situation, each sensor is regarded as a random variable with two results, “ON” and “OFF”. Krishnan and Cook defined the mutual information or dependency between two sensors as the possibility that these two sensors appear consecutively throughout the sensor event sequence [33]. This definition is affected by the order in which each pair of sensors appears in the whole dataset. Consider deploying four sensors involving a specific activity in a tight place, and the resident can adopt the route that triggers sensors in the order of $S_{1}\to S_{2}\to S_{3}\to S_{4}$, or in another way, $S_{1}\to S_{2}\to S_{4}\to S_{3}$, to implement this activity. It is assumed that the number of the first path is greater than that of the second one, but these two paths point to the same human activity; it is evident that no matter which path is taken, there is a dependency between sensors $S_{2}$ and $S_{3}$. If the above definition method is adopted to calculate the mutual information between $S_{2}$ and $S_{3}$, some dependencies between them will be lost. Therefore, we adopt its extension definition and calculate mutual information between two sensors $S_{i}$ and $S_{j}$ by calculating the probability that they co-occur in a sliding window with ws sensor events and a sliding length of 1 along the entire data stream. Let $W=\left\{\ldots ,W_{k},\ldots \right\}$ represent the segmented sliding window sequences, where $W_{k}$ denotes the *k*th window of the streaming dataset; then, the mutual information $MI(S_{i},S_{j})$ is defined as follows:

(1) $$\left\{\begin{array}{c} MI\left(S_{i},S_{j}\right)=\frac{1}{\left|W\right|}\sum_{k=1}^{\left|W\right|}\delta (S_{i},S_{j})\\ \delta \left(S_{i},S_{j}\right)=\left\{\begin{array}{c} 1,\;\mathrm{if}\;\left(S_{i},S_{j}\right)\in W_{k}\\ 0,\;\mathrm{else} \end{array}\right. \end{array}\right.$$

where $\delta (S_{i},S_{j})$ takes 1 when sensors $S_{i}$ and $S_{j}$ co-occur in a sliding window; $MI(S_{i},:)$ denotes the dependence of sensor $S_{i}$ with other sensors. The mutual information matrix (i.e., SCM) is a symmetric matrix and calculated offline using the training sensor event sequence.

After obtaining SCM, we will determine the SCT value for each sensor based on it. Typically, a specific activity activates sensors in the corresponding functional area and generates sensor events. Obviously, SCM values between sensors with smaller spatial distance are more likely to be greater, indicating that there is a higher probability to activate the sensors in the same or close functional areas jointly or consecutively. Therefore, we perform the following steps for each row of SCM (corresponding to one sensor) to obtain the corresponding SCT value: first, sort the row in descending order, and then combine with the layout of sensors in the smart home to find the sensor that is geographically critical to the current sensor and its SCM value that is used as the minimum sensor correlation threshold of the current sensor. As a result, given an interested sensor $S_{i}$ and the sensor sequence $S=\left\{S_{1},\ldots ,S_{n}\right\}$, if the SCM value between $S_{i}$ and $S_{j}$ is greater than the SCT(i) value, it can be considered that sensors $S_{i}$ and $S_{j}$ are spatially correlative.

#### 3.1.2. Time Correlation

In the dynamic segmentation approach, in addition to measuring the sensor correlation, on the other hand, it also has to accurately determine whether two sensor events with some time interval should be placed in the same window. For instance, given two sensor events in which the dependency between the two sensors may be high, but the time interval may be very large, then these two sensor events should not be in the same sliding window. In consequence, a measure based on time correlation is employed to judge whether two sensor events are temporally dependent. As mentioned earlier, a sequence of sensor events can be represented as $\left\{E_{1},\;E_{2},\ldots ,E_{N}\right\}$, where each $E_{i}\in E$ contains the information vector $\langle D_{i},T_{i},S_{i},V_{i}\rangle$. $D_{i}$ and $T_{i}$ denote the date and timestamp of the sensor event, respectively, $S_{i}$ signifies the sensor ID or name, and $V_{i}$ denotes the value of $S_{i}$. Assuming that a partial segment $W_{i}=\left[E_{first},\ldots ,E_{i-1},E_{i}\right]$ of the latest recorded sensor event $E_{i}$ has been selected, then each incoming sensor event $E_{first-1}\in E$ is manipulated twice with $T_{first}$ and $T_{i}$ utilizing Equations (2) and (3), respectively.

(2) $$T_{first}^{cor}\left(T_{first-1},T_{first}\right)=1-\frac{T_{first}-T_{first-1}}{MTI(S_{first-1},S_{first})}$$

(3) $$T_{i}^{cor}\left(T_{first-1},T_{i}\right)=1-\frac{T_{i}-T_{first-1}}{MTS(f\left(S_{i}\right))}$$

where the threshold $MTI(T_{first-1},T_{first})$ is defined according to the distribution of the time interval between sensors $S_{first-1}$ and $S_{first}$ in two consecutive sensor events $E_{first-1}$ and $E_{first}$. In consideration of the very small proportion of points away from the average time interval and the “2σ” criterion, we take $\mu \left(S_{first-1},S_{first}\right)+2\sigma \left(S_{first-1},S_{first}\right)$ as the value of threshold $MTI(T_{first-1},T_{first})$, where $\mu (S_{first-1},S_{first})$ and $\sigma (S_{first-1},S_{first})$ denote the mean and standard deviation, respectively. The sensor event sequence in the dataset is recorded in chronological order, so $T_{first}-T_{first-1}>0$ and $MTI(S_{first-1},S_{first})>0$. With regard to the threshold $MTS(f\left(S_{i}\right))$, it is related to the duration of activities. As mentioned earlier, in the smart home environment, the layout of each sensor corresponds to the functional area. Combined with SCM, the functional areas of the testbed used in this paper can be further clustered into five areas: ① *Kitchen+Dinning*, ② *Bedroom+Bathroom*, ③ *Living*, ④ *Office*, ⑤ *Home Entrance*. Through studying the long-term activity data of *Meal_Preparation*, *Eating* and *Wash_Dishes*, 88.10% of the sensor firings appear in the *Kitchen+Dinning* area; 88.01% of sensor events of *Relax* appear in the *Living* area. In addition, 94.57% of sensors for activity *Work* are triggered in the *Office*, and 93.69% sensor events for activities of *Enter_Home* and *Leave_Home* occur in the *Home Entrance* area. Furthermore, 97.08% sensor activations for *Sleeping* and *Bed_to_Toilet* take place in the area of *Bedroom+Bathroom*. On the basis that the resident keeps a relatively regular schedule, assuming that daily activities will not change significantly, different threshold settings of activity duration and the sensing range of sensors can be operated according to the clustered functional areas. In the same way as determining $MTI(T_{first-1},T_{first})$, the “2σ” point of the distribution of the duration of activities in each clustered functional area is also used as the threshold of the activity duration of each clustered functional area, as described in Equation (4). Specifically, for partial segment $W_{i}=\left[E_{first},\ldots ,E_{i-1},E_{i}\right]$, first determine the mapping from the sensor $S_{i}$ to the clustered functional area, and then obtain the corresponding MTS value $MTS(f\left(S_{i}\right))$. Likewise, $T_{i}-T_{first-1}>0$ and $MTS(f\left(S_{i}\right))>0$.

(4) $$\left\{\begin{array}{c} MTS\left(f\left(S_{i}\right)\right)=\mu \left(f\left(S_{i}\right)\right)+2\sigma \left(f\left(S_{i}\right)\right)\\ f\left(S_{i}\right)\in \left\{1,2,3,4,5\right\} \end{array}\right.$$

To summarize, Algorithm 1 shows the pseudo code of the dynamic sensor data segmentation method. This algorithm processes real-time streaming sensor data, which comprises two important comparisons: the sensor correlation check (SCC) and the time correlation check (TCC). For the currently recorded sensor event $E_{i}$, the window is initialized to $W_{i}=\left[E_{i-1},\;E_{i}\right]$ and $T_{first}=T_{i-1}$. Thereafter, both SCC and TCC will be conducted for sensor event $E_{first-1}$, which will be added to the segmentation if the check results align with Equation (5). Otherwise, the current segmentation process ends.

1.6

(5) $$\left\{\begin{array}{c} SCM\left(S_{first-1},S_{i}\right)\geq SCT\left(S_{i}\right)\\ T_{first}^{cor}\left(T_{first-1},T_{first}\right)\geq 0\\ T_{i}^{cor}\left(T_{first-1},T_{i}\right)\geq 0 \end{array}\right.$$

**Algorithm 1** Dynamic sensor data segmentation method.

**Input:**

Streaming sensor data: $E=\left\{E_{1},E_{2},\cdots ,E_{i-1},E_{i}\right\}$
Initialization window for the sensor event: $W_{i}=\left[E_{i-1},E_{i}\right]$, $E_{first}=E_{i-1}$

**Output:**

A sensor event segmentation for $E_{i}$: $W_{i}=\left[\cdots ,E_{i-1},E_{i}\right]$

**Method:**

  Sensor correlation check (SCC): $SCM\left(S_{j},S_{i}\right)\geq SCT\left(S_{i}\right)$
  Time correlation check (TCC): $T_{first}^{cor}\left(T_{j},T_{first}\right)\geq 0$, $T_{i}^{cor}\left(T_{j},T_{i}\right)\geq 0$
  **for** $E_{j}$ From $E_{i-2}$ To $E_{1}$ **do**
    **if** $SCM\left(S_{j},S_{i}\right)\geq SCT\left(S_{i}\right)$ && $T_{first}^{cor}\left(T_{j},T_{first}\right)\geq 0$ && $T_{i}^{cor}\left(T_{j},T_{i}\right)\geq 0$ **then**
      $W_{i}=\left[E_{j},W_{i}\right]$
      $E_{first}=E_{j}$
    **else**
      Break
    **end if**
  **end for**



It is worth noting that different smart home environments are deployed with a variety of sensors to collect the information of ADLs, and even within the same smart home, different users perform each activity in their unique ways. Our proposed AR framework only needs to acquire SCM, SCT, MTI and MTS as prior knowledge for dynamic segmentation according to the layout of the smart home and the characteristics of the personalized training data. Except for this, the proposed online AR method can be generalizable well to be reusable for different application scenarios.

### 3.2. Activity Modeling

Once the window $W_{i}$ of sensor event $E_{i}$ is determined, then we need to convert this segmented window into features that capture the spatio-temporal information to define the context of $E_{i}$. Some works in the literature accomplish this process by establishing a feature vector $x_{i}$ that explicitly captures the activation duration of each sensor. There are 31 motion sensors and four door sensors in the context of the testbed used in this study; consequently, the dimension of $x_{i}$ is 35 and it can be expressed as $x_{i}=\left[d_{1}^{i},\;d_{2}^{i},\cdots ,d_{35}^{i}\right]$. Attach a label to every $x_{i}$ with the tag $y_{i}$ of the last event $E_{i}$ in the corresponding window $W_{i}$, and each label $y_{i}$ corresponds to a predefined activity category.

There are some disadvantages in simply counting or accumulating the activation time of each sensor. On the one hand, the order of the triggered sensors is not reflected; that is, the coarse trajectory of the resident cannot be well represented. On the other hand, the timeliness of information is not taken into account in the features extracted to define the context of the last sensor event in the window. In this case, the sensor data occurring in the "distant" past have the same impact as recent sensor events. However, generally, the more recent the sensor event occurs, the more information it can provide, and vice versa. We adopt the emergent paradigm with marker-based stigmergy for activity modeling so as to overcome the above limitations. The emergent paradigm is based on the principle of the self-organization of data. With the emergent modeling method, the focus is mainly on the low-level data processing, which enables aggregation perception in the environment. Furthermore, the overall characteristics of the sensor event sequence can be described with a domain-independent spatio-temporal logic.

It is well known that simple individual behaviors can give rise to complex emergent behaviors. In natural systems, social insects use chemical markers (pheromones) released on the ground in specific circumstances, such as assembling, foraging or alarming. Multiple deposits at the same position accumulate in intensity. Individuals in a group may change their behaviors after sensing a particular pheromone. Due to their high volatility, the intensity of the released pheromones gradually decreases over time. In artificial systems, when markers are produced in the computer-simulated spatial environment, marker-based stigmergy will appear to realize self-coordination and self-organization, which would be regarded as a potential computing paradigm using spatial and temporal dynamics.

In the process of activity modeling, we take advantage of stigmergy as a pattern of information aggregation for human spatio-temporal tracks, where the process of information aggregation is an abstract vehicle that leads to the emergence of a higher-level concept. When the sensors are triggered by the human motion, the corresponding marks, with a temporal decay called the volatilization rate $\rho \in \left[0,1\right]$, i.e., a ratio of reduction after a time step, will be continuously released in the environment, which can realize the accumulation of marks. Therefore, a separate mark after a certain amount of time will vanish because there is no new mark to strengthen its concentration, and the aggregated mark, i.e., stigmergic track, considered as a short-term and short-size motion memory, can intuitively embody the spatio-temporal characteristics.

**Figure 3:**
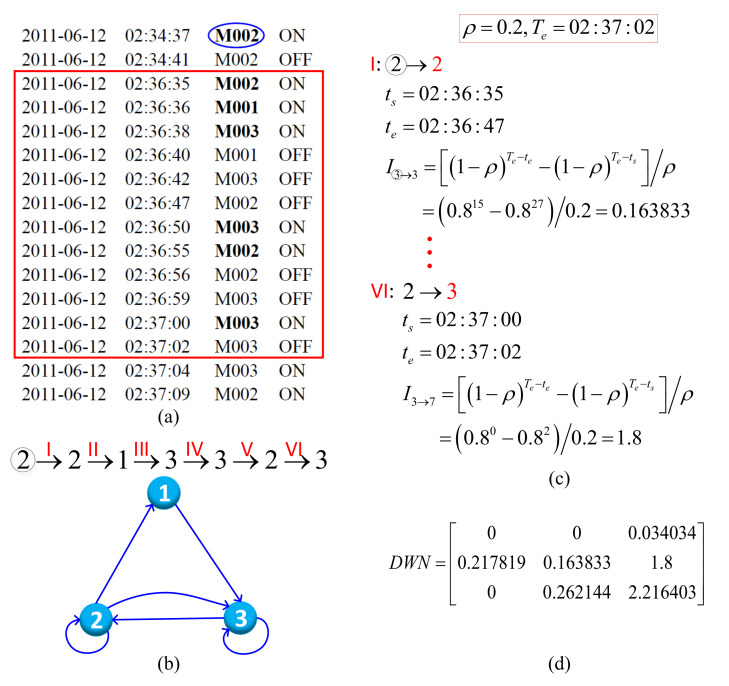
The process from segmented window to DWN. (**a**) Segmented sensor data. (**b**) Directed weighted network. (**c**) Intensity of aggregated pheromone. (**d**) Adjacency matrix of DWN.

In this paper, DWN and its corresponding adjacency matrix are used to represent the stigmergic track vividly and explicitly. Figure 3 illustrates an example of the generation process from segmented data sequence to DWN, where ρ=0.2. The selected window is a segment framed by a red rectangle in Figure 3a.

Owing to the fact that the passive sensors are triggered by the motion of the resident, the position of the activated sensor can be approximately regarded as the location of the resident at the current moment. First, we extract the triggered sensors and their triggered order: ②→2→1→3→3→2→3. The corresponding DWN is shown in Figure 3b, where the sensor that is triggered before the first activated sensor “M002” in the window is also “M002”, so we obtain the self-loop “②→2”. The remaining directed edges can be easily obtained according to the trigger order. Then, we calculate the weight of each directed edge (including self loops), i.e., the concentration of the pheromones deposited by the sensor corresponding to the right side of the arrow of the directed edge. In each time period, one unit of pheromone is released at the corresponding position in the environment of the triggered sensor, and the pheromone concentration reduces with volatilization rate ρ after a time step. Here, we set a time step to 1s. For the segmented window $W_{i}$, the end time $T_{e}$ is equal to the time of the last sensor event $E_{i}$, i.e., $T_{e}=T_{i}$, and $T_{i}=02:37:02$ in this example. For each directed edge, the trigger start time $t_{s}$ and trigger end time $t_{e}$ of the related sensor are extracted. Without time volatility, the intensity of aggregated pheromones is equal to the activation duration $t_{e}-t_{s}$. When time volatility is introduced, the intensity of pheromones *I* is calculated as shown in Equation (6), which describes the superposition of the newly generated pheromone and the volatilized old pheromones.

(6) $$\begin{array}{c} I=\sum_{t=t_{s}}^{t_{e}-1}{\left(1-\rho \right)}^{T_{e}-t-1}\\ \;=\frac{{\left(1-\rho \right)}^{T_{e}-t_{e}}-{\left(1-\rho \right)}^{T_{e}-t_{s}}}{\rho } \end{array}$$

For instance, as displayed in Figure 3c, the start and end time of self-loop “②→2” are “02:36:35” and “02:36:47”, respectively. The concentration of the aggregated pheromone of this directed edge is I2→2=0.815−0.8270.815−0.8270.20.2=0.163833. The weight of the last edge “2→3” is: I2→3=0.80−0.820.80−0.820.20.2=1.8. With the same calculation method, we can obtain the concentrations of all aggregated pheromones, so as to obtain the corresponding adjacency matrix (Figure 3d) of the directed weighted network, which describes the spatio-temporal traits of the segmented window and defines the context of the last sensor event.

For convenience, the directed weighted network and adjacency matrix are collectively referred to as “DWN” in the following. From DWN, we can not only know the intensity of pheromones released when each sensor is activated, but also know the location of the resident before the sensor is triggered. That is, the explicit representation method of DWN can distinguish different pheromone sources’ information and deduce a coarse stigmergic track. Furthermore, with the emergent computing paradigm with marker-based stigmergy, the concentration of the old pheromones gradually decreases over time due to the volatility, and the effect on the context that defines the last sensor event is gradually weakened, while the influence of the new pheromones is relatively greater. This also reflects the limited memory characteristics of context-aware information, the trigger sequence information of sensors and the motion process information of the resident. In our method, DWN is used as the input of the fine-grained classification algorithm.

### 3.3. Fine-Grained Classification

After activity modeling, the fined-grained classification algorithm takes DWN as the input and outputs the category of activity whenever the new sensor event is recorded. We employ a method that combines CNN and LSTM for online AR on streaming sensor data. The CNN plays the role of a slice-wise feature extractor that selects the most effective features from input data, while the LSTM, a powerful tool for learning the sequential task, is responsible for linking the features across slices.

## 4. Experiments

In this section, to evaluate the proposed online activity recognition method combined with the spatio-temporal correlation-based dynamic streaming sensor data segmentation approach and the stigmergy-based emergent modeling approach, we employ the open dataset Aruba provided by CASAS [13]. We describe the dataset, performance measurements and experimental studies.

### 4.1. Dataset

**Figure 4:**
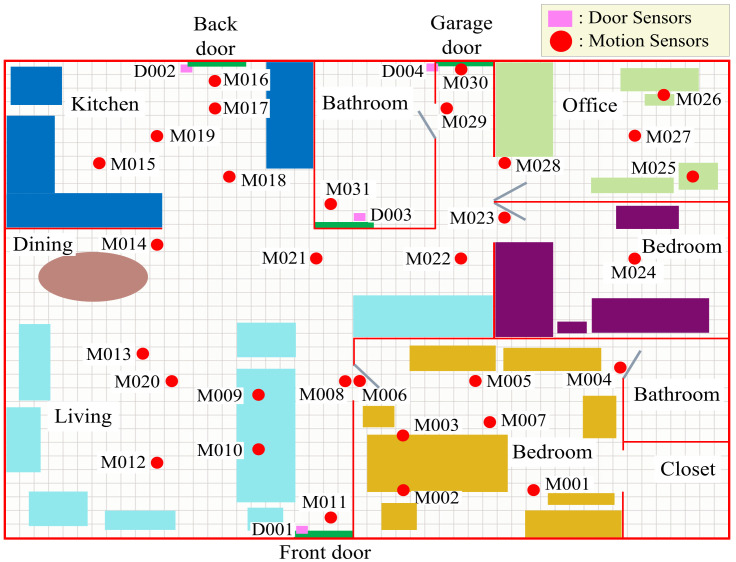
The layout of Aruba and locations of sensors.

**Figure 5:**
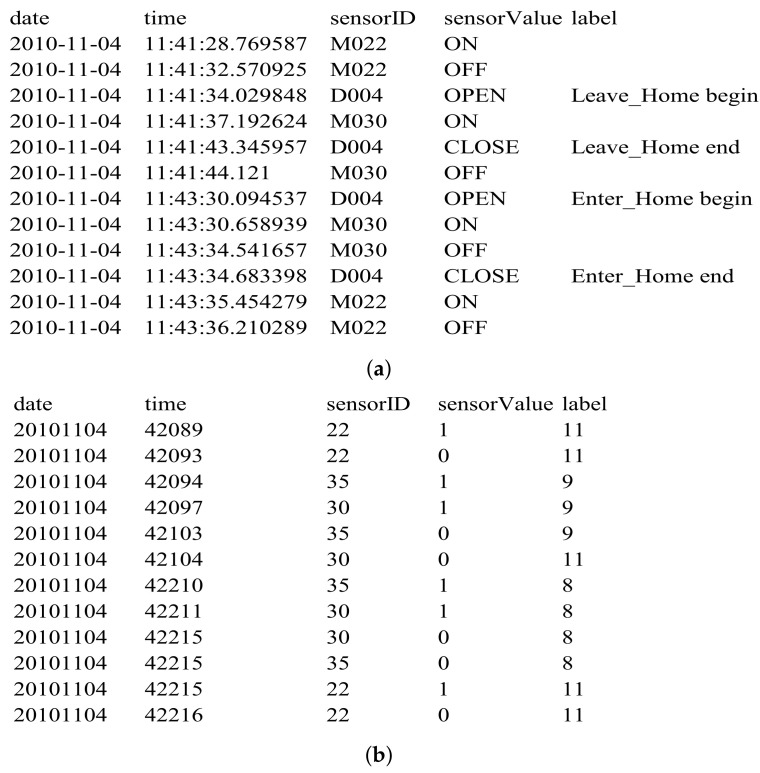
A sample dataset of Aruba. (**a**) Raw data sample. (**b**) Digitized data sample.

The Aruba dataset was collected by detecting ADLs for an elderly woman in a smart home for nearly eight months, which includes annotated binary sensor data. The layout of the smart home and the positions of sensors are presented in Figure 4. This testbed consists of two bedrooms, a living room, a kitchen, a dining room and an office, which is embedded with 31 wireless motion sensors (named with “M0**”) installed on the ceilings, four door sensors (named with “D0**”) installed on the door frames and four temperature sensors. Only the information of binary sensors (motion and door sensors) is adopted in this work, because the temperature sensors cannot provide the explicit motion process information of the resident. Therefore, the size of DWN is 35×35. Figure 5a represents a sample of the dataset, where the annotated sensor events in the dataset include ten categories of predefined activities of daily living, while the untagged sensor events are all labeled with “Other Activity”. The number of sensor events for each class of ADLs in the whole dataset is displayed in Table 1, which shows that the number among different activities varies greatly and the “Other Activity” with more than 50% sensor events dominates the dataset. For convenience, the dataset is digitized. Concretely, the 11 categories of activities from “Meal_Preparation” to “Other Activity” are mapped into the integers “1–11”, respectively. The motion sensors and door sensors are converted to “1–31” and “32–35”, respectively, whose “SensorValue” of “ON/OPEN” and “OFF/CLOSE” are mapped to “1” and “0”, respectively. “Date” is converted to the form of “yyyymmdd”, and “Time” is converted to the timestamp (in seconds) relative to the zero hour of the current day. The digitized data sample is shown in Figure 5b.

### 4.2. Evaluation Measures

Because the focus is to evaluate the overall performance of all activities including “Other Activity” when different methods are employed, we compute the accuracy and F1 score. For binary classification, true positive (TP), false negative (FN), true negatives (TN) and false positive (FP) shown in Table 2 are calculated, and the precision *P* and recall *R* can be defined by Equations (7) and (8).

(7) $$P=\frac{TP}{TP+FP}$$

(8) $$R=\frac{TP}{TP+FN}$$

For computing weighted measures, (P,R) pairs of each category activity are calculated: $\left(P_{1},R_{1}\right),\;\left(P_{2},R_{2}\right),\ldots ,\left(P_{\left|A\right|},R_{\left|A\right|}\right)$, where $\left|A\right|$ denotes the number of categories for all activities including “Other Activity”, and $N_{j}$ represents the number of sensor event windows relevant to a kind of activity *j*. Then, the weighted mean, $\bar{P}$ and $\bar{R}$, can be obtained. Therefore, the accuracy Acc and weighted F1 score F1 are calculated as in Equation (9):

(9) Acc=∑j=1ATPj∑j=1ATPj∑j=1ANj∑j=1ANjF1=2·P¯·R¯2·P¯·R¯P¯+R¯P¯+R¯

### 4.3. Experimental Results

We use the CNN-LSTM architecture as the fine-grained classifier to learn the activity classification model. The segmentation methods on streaming sensor data include the event-based segmentation method that takes a fixed window size (we refer to this method as *FS*) and the proposed dynamic segmentation method that adopts a dynamic window size (referred as *DS*). The activity modeling approaches include the feature vector approach (referred to as *FV*) and the DWN approach. We employ the five-fold cross-validation strategy and the offline phase is performed on the training dataset to compute SCM, SCT, MTI and MTS according to Section 3.1.

**Figure 6:**
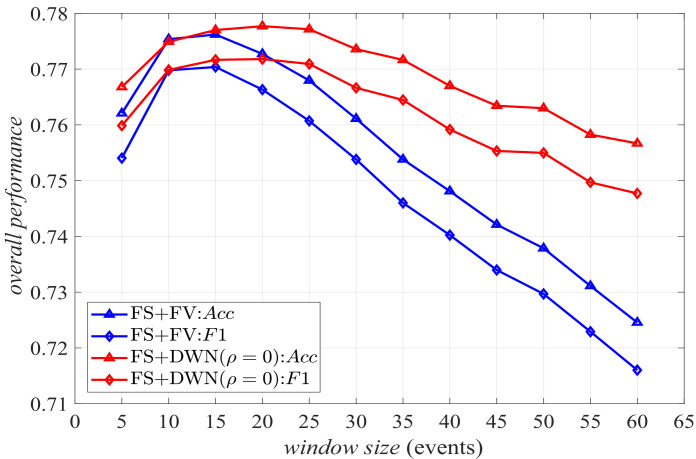
Overall performance of *FS*+*FV* and *FS*+DWN (ρ=0).

Figure 6 shows the results employing *FS*, in which the window size increases from 5 to 60 sensor events, and the activity modeling adopts *FV* and DWN without volatility (i.e., ρ=0), respectively. It is easy to see that the performance of both *FV* and DWN with ρ=0 first increases and then decreases with the increase in window size ws. They achieve the best overall performance when ws=15 and ws=20, respectively. When the number of sensor events per window is small, there is insufficient information to define the context of the last sensor event in the window. On the contrary, a large window size leads to a lot of redundant information. The above two cases are unfavorable to accurately describe the activity features, so the overall performance is not good. Furthermore, when a specific window size is given, the results of DWN are better than those of *FV*. Compared with the feature vector, the representation of the directed weighted network can not only obtain the activation duration of each sensor, but also obtain the location information of the resident before each sensor is triggered, which reflects the rough motion process.

**Figure 7:**
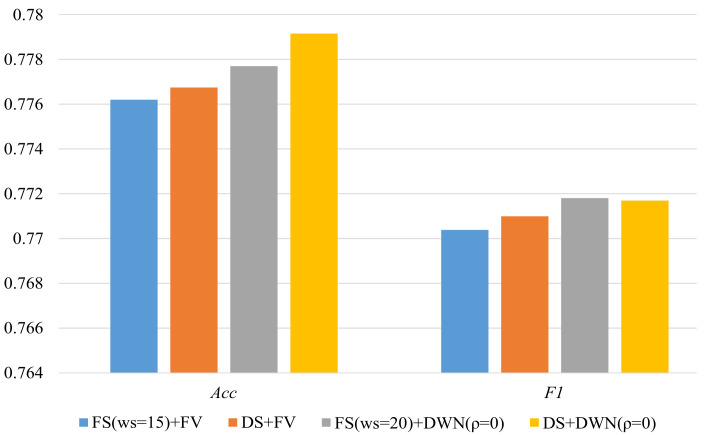
Classification performance of *FS* and *DS*.

Next, we use dynamic segmentation approach to determine the sliding window for each sensor event when it is recorded, and then use the above two activity representation methods to perform activity modeling. The results are shown in Figure 7, which illustrate that the dynamic segmentation approach obtains better classification performance than the fixed window size approach.

**Figure 8:**
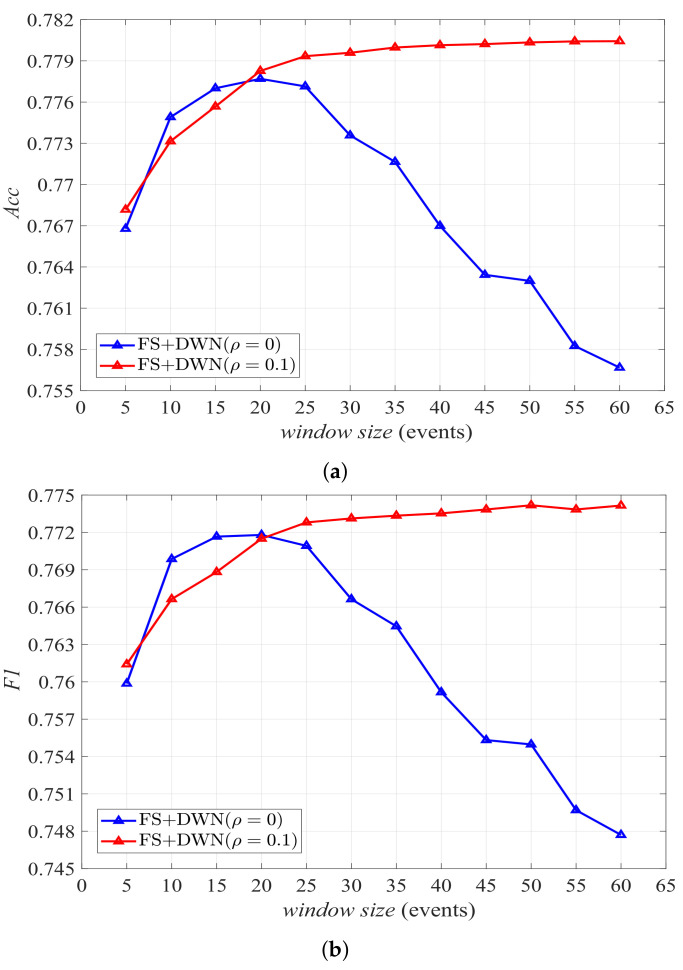
Results of employing *FS*+DWN with ρ=0 and ρ=0.1, respectively. (**a**) Accuracy. (**b**) F1 score.

The results of employing *FS* and DWN with volatility (ρ=0.1) are displayed in Figure 8. Different from *FV* and DWN without volatility, the accuracy and F1 score in this case gradually increase as the window size increases, and finally converge to a stable value that is greater than the maximum value obtained at ws=20 when there is no volatility. Activity modeling based on the emergent paradigm with marker-based stigmergy can reduce the impact of sensor data far away from the interested sensor event on the context for defining the last sensor event in the segmented window, so as to reduce redundant information. In contrast, the sensor events in the sliding window that are closer to the last event provide more information for defining the context. Therefore, when the window size increases to a certain value, the context information provided by the “distant” past triggered sensor events is very little, resulting in the overall classification performance converging to a stable state.

**Figure 9:**
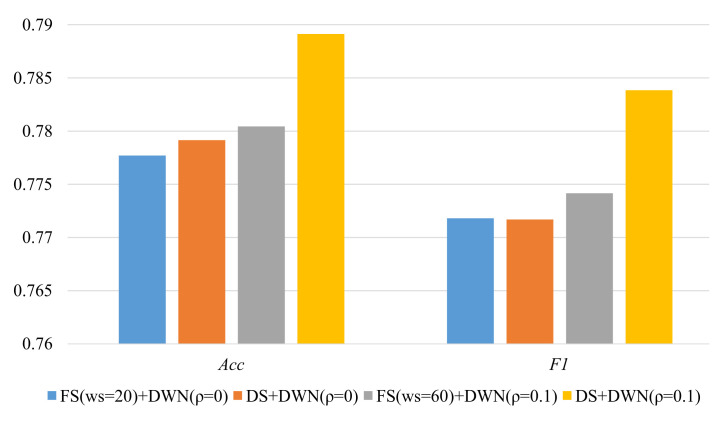
Comparison results of volatility and non-volatility, fixed and dynamic window size.

The proposed online activity recognition method on streaming sensor data integrates the dynamic segmentation approach and the emergent modeling method. Figure 9 displays the overall performance of the following four cases: ① *FS*+DWN without volatility, ② *DS*+DWN without volatility, ③ *FS*+DWN with volatility and ④ *DS*+DWN with volatility. We can easily find that the results of the proposed fusion method integrating spatio-temporal correlation-based dynamic windowing and stigmergy-based emergent modeling are obviously better than the other three cases.

**Figure 10:**
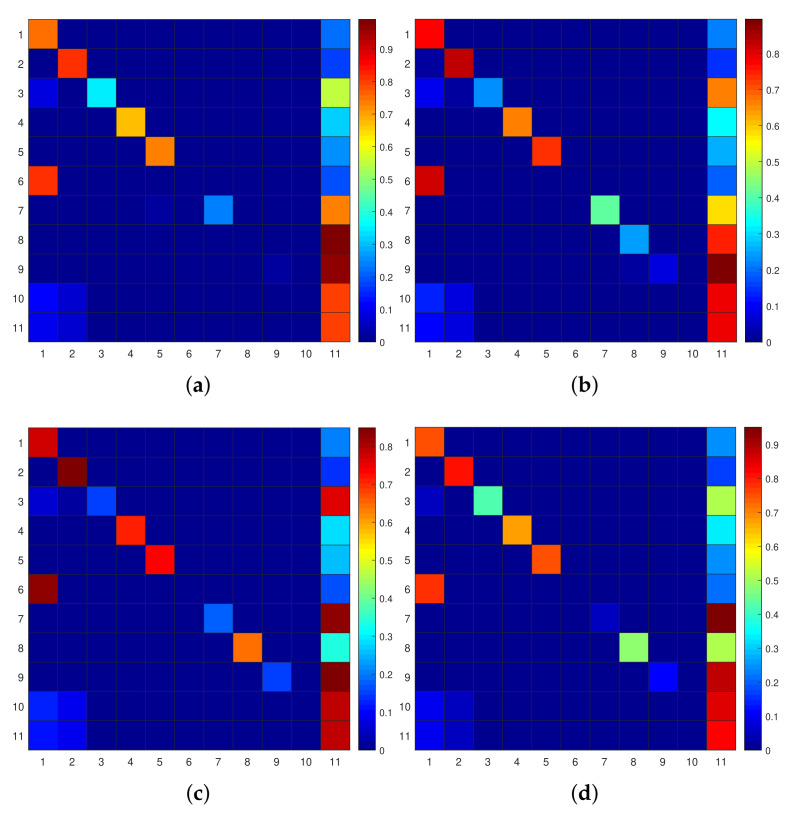
Confusion matrixes using different approaches. (**a**) *FS* (ws=15) + FV. (**b**) *FS* (ws=20) + DWN (ρ=0). (**c**) *FS* (ws=60) + DWN (ρ=0.1). (**d**) *DS* + DWN (ρ=0.1).

In addition to studying the overall performance, we also explore the classification performance of different categories of activities in various cases discussed earlier, and their confusion matrixes are shown in Figure 10. We observe that “Enter_Home” and “Leave_Home” activities benefit the most by employing the emergent paradigm with marker-based stigmergy. The improvement for these two activities is understandable as they have clear directionality and a well-defined past context. However, on the other hand, “Meal_Preparation” and “Wash_Dishes” have high confusion because they share the same functional area and corresponding deployed sensors. Moreover, they are not as directional as “Enter_Home” and “Leave_Home” and the number of segmented windows related to “Wash_Dishes” is far lower than that of “Meal_Preparation” (0.6524% vs. 18.0637%), which leads to the poor classification performance of “Wash_Dishes”. Another obvious confusion is included in “Other Activity”. As can be seen from the confusion matrixes, many sensor data belonging to the predefined ten ADLs are misclassified as “Other Activity”. There are several reasons for this performance change. On the one hand, more than 50% of the sensor events in the dataset belong to “Other Activity”, which dominates the entire dataset, resulting in a number of data belonging to predefined activities being identified as “Other Activity”. On the other hand, “Other Activity” itself may be mixed with a variety of different activities, transitions between activities and movement patterns, which makes it difficult to characterize this complex class and distinguish it well from the other predefined activities.

Finally, the proposed online activity recognition method is compared with several existing methods:

*SWMI*: It employs a constant window size, in which each window has the same number of sensor events. The mutual information between two sensors defined as the probability of these two sensors appearing consecutively in the whole data stream is taken into account when extracting features [33].

*SWMIex*: The only difference between this method and *SWMI* is that it defines mutual information as the possibility that two specified sensors arise simultaneously in one window of the entire dataset [37,38].

*SW*: This model utilizes sensor event-based windowing and every sensor event is equally contributing in the feature vector [33].

*SWTW*: There are the same number of sensor events in each window in this model, and it uses a time-based weighting factor to calculate the contributions of each sensor event to the feature vector [33].

*SWMI+SWTW*: This method combines *SWMI* and *SWTW*.

*TW*: It adopts an equal time interval to divide the entire sensor event sequence into a series of segments [33].

**Figure 11:**
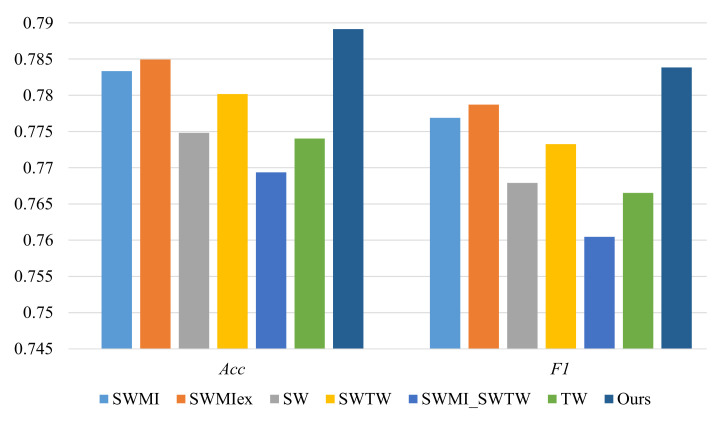
Performance using different online AR models.

All the classifiers adopt the CNN-LSTM architecture and the corresponding results are shown in Figure 11, which demonstrates that our proposed model obtains better performance than the other commonly used models. This mainly due to the following reasons. On the one hand, as far as the window segmentation is concerned, in order to provide accurate context for the latest sensor event as much as possible, the sensor events contained in the segmented window should be correlative to the target event in time and space. However, sensor event-based or time-based windowing methods simply and roughly divide the sensor data stream into a set of sequences, resulting in the segmented windows that either contain too much redundant information or too little effective information, both of which are not conductive to defining the context and accurately identifying the corresponding activity being performed. In contrast, our proposed dynamic segmentation method takes into account the spatio-temporal correlation of sensor events in the window, ensuring that events with a large time interval or from very different functional areas will not be placed in the same window. On the other hand, in terms of activity modeling, the extracted shallow information (such as the trigger times or trigger duration of sensors) cannot reflect the context information well and loses some hidden information. The stigmergic tracks obtained based on the emergent modeling method can not only reflect the duration of residents staying at each position, but also roughly characterize the motion process. In addition, the impacts of sensor events from the “distant” past on the definition of the context about the interested event can be weakened by exploiting volatility. Both window segmentation and activity modeling approaches are critical to determine the ongoing activity, so the combination of these two aspects can result in good performance. In summary, the results of both ablation experiments and comparative experiments verify the effectiveness of the online activity recognition model that integrates the emergent modeling method based on stigmergy and the dynamic segmentation method considering sensor correlation and time correlation.

## 5. Conclusions

This paper presents an online activity recognition model on streaming sensor data for monitoring elderly behavior. The online AR method combines the spatio-temporal correlation-based dynamic segmentation approach and the stigmergy-based emergent modeling approach to recognize the ongoing activity when a new sensor event is recorded. The dynamic segmentation method integrates sensor correlation and time correlation to estimate whether two consecutive sensor events belong to the same window or not, avoiding sensor events from very different functional areas or with a long time interval in the same window, so as to determine the final segmented window for every individual sensor event. After this, the emergent paradigm with marker-based stigmergy is employed to establish activity features by aggregating sensor data at the low level for defining the context of the last sensor event in the segmented window. This activity modeling method is domain knowledge-independent, and it adopts the directed weighted network that can distinguish different pheromone sources to explicitly represent the extracted features. With the temporal volatility of pheromones, the aggregated marks can reduce the impact of early sensor events on the context, and recent sensor events play a relatively greater role, which reflects the limited memory characteristics of context information. The open dataset Aruba offered by CASAS is employed to evaluate the effectiveness of our model. The ablation experiments show that the results of adopting *DS*+DWN with volatility are superior to those of the other cases. Moreover, the overall performance of the proposed method is shown to be better than that of the existing methods in comparative experiments. All of the above demonstrate the effectiveness of the proposed online activity recognition method integrating the dynamic segmentation and emergent modeling.

## Figures and Tables

**Table 1:** Number of sensor events of activities.

Activity Name	Number of Events	Proportion (%)
1-Meal_Preparation	288,407	18.06370999
2-Relax	347,911	21.79060635
3-Eating	16,352	1.02416996
4-Work	16,321	1.022228346
5-Sleeping	32,535	2.037754993
6-Wash_Dishes	10,417	0.652444868
7-Bed_to_Toilet	1310	0.082048841
8-Enter_Home	2003	0.125453304
9-Leave_Home	1914	0.119878994
10-Housekeeping	10,579	0.662591365
11-Other Activity	868,861	54.419113

**Table 2:** The confusion matrix.

Confusion Matrix		Predicted Result	
		Positive	Negtive
**True Result**	True	True Positive (TP)	False Negtive (FN)
	False	False Positive (FP)	True Negative (TN)

## Data Availability

Aruba dataset is available at: http://casas.wsu.edu/datasets/ (accessed on 31 January 2022).
